# Stem Cell Therapy: A Compassionate Use Program in Perianal Fistula

**DOI:** 10.1155/2019/6132340

**Published:** 2019-05-05

**Authors:** M. D. Herreros, D. Garcia-Olmo, H. Guadalajara, T. Georgiev-Hristov, L. Brandariz, M. Garcia-Arranz

**Affiliations:** ^1^Department of Surgery, University Hospital Fundación Jiménez Díaz, Madrid, Spain; ^2^New Therapy Laboratory, Instituto de Investigación Sanitaria Fundación Jiménez Díaz, Madrid, Spain; ^3^Surgery Department, Universidad Autonoma de Madrid, Madrid, Spain; ^4^Department of Surgery, Hospital General de Villalba, Collado Villalba, Madrid, Spain

## Abstract

**Aim:**

To report our experience in a compassionate use program for complex perianal fistula.

**Methods:**

Under controlled circumstances and approved by European and Spanish laws, a compassionate use program allows the use of stem cell therapy for patients with nonhealing diseases, mostly complex fistula-in-ano, who do not meet criteria to be included in a clinical trial. Candidates had previously undergone multiple surgical interventions that had failed. The intervention consisted of surgery (with closure of the internal opening or a surgical flap performance), followed by stem cell injection. Three types of cells were used for implant: stromal vascular fraction, autologous expanded adipose-derived, or allogenic adipose-derived stem cells. Healing was evaluated at 6^th^ month follow-up. Outcome was classified as partial response or healing. Relapse was evaluated 1 year later. Maximum follow-up period was 48 months.

**Results:**

45 patients (24 male) were included; the mean age was 45 years, which ranged from 24 to 69 years. Since some of them received repeated doses, 52 cases were considered (42 fistula-in-ano, 7 rectovaginal fistulas, 1 urethrorectal fistula, 1 sacral fistula, and 1 hidradenitis suppurativa). Regarding fistula-in-ano, there were 18 Crohn's-associated and 24 cryptoglandular. 49 cases (94.2%) showed partial response starting 6.5 weeks of follow-up. 24 cases (46.2%) healed in a mean time of 5.5 months. A year later, all patients cured remained healed. No adverse effects related to stem cell therapy were reported.

**Conclusion:**

Stem cells are safe and useful for treating anal fistulae. Healing can be achieved in severe cases.

## 1. Introduction

The incidence of fistula-in-ano ranges from 1.1 to 2.2 per 10,000 population per year [1, 2]. Although most cases are easily treated by surgery, management of “complex fistulas” remains a challenge [3–5]. Limited surgery may result in recurrence while aggressive surgery is associated with fecal incontinence [3].

Application of autologous adipose-derived stem cells (ASCs) represents a novel approach for enhancing regeneration and/or repair of damaged tissues [6, 7] in an environment particularly unfavorable for wound healing [8–11]. It has been hypothesized that the immunoregulatory and anti-inflammatory properties of ASCs may work together to accelerate healing [12, 13]. ASCs are purified from adipose tissue [14, 15], which can be readily and safely obtained by liposuction [11]. The process yields 100 times more stem cells than bone marrow aspirates [16] and the process is considered safe [17].

A previous proof-of-concept phase I clinical trial in fistulizing Crohn's disease [18] and a phase II and two phase III clinical trial (CT) in fistulas of cryptoglandular origin and fistulas associated to Crohn's disease [19–21] found ASCs to be safe and effective.

Since March 2018, allogenic stem cells are available and ready to be marketed for patients with Crohn's disease, authorized by EMA (European Medicine Agency) as Alofisel® (darvadstrocel). Alofisel® indications are complex perianal fistula in Crohn's disease with low or mild bowel activity, not responding to medical treatments.

Our institution is an international referral center. We developed a compassionate use program in order to expand the stem cell therapy to all the patients who can benefit from it. Criteria to be included in this program were

- patients who did not meet criteria of the clinical trial (CT) in development
- foreign patients, who were not allowed to be included in the CT
- patients included in some CT control arms
- failure treatment in patients included in a CT treatment arm as a retreatment

The aim of this paper is to report our experience in a compassionate use program since April 2014 to nowadays in patients with at least 6 months of follow-up.

## 2. Materials and Methods

We present an observational study, including 52 cases in 45 patients (24 male and 21 female) treated by a compassionate use ASC program. From now on, we will refer to cases instead to individual patients because some of them had several ASC injections. The mean age was 45 years and ranged from 24 to 69 years. There were 42 perianal fistulas, 7 rectovaginal fistulas, 1 urethrorectal fistula, 1 sacral fistula, and 1 hidradenitis suppurativa. All the cases had previous failed surgeries. Thirteen of the fifty-two (25%) cases presented fecal incontinence at the moment of enrollment in this study but only 7 cases completed a Wexner Score [22]. 5/52 (9.6%) of the cases presented anal stenosis and 11/52 (21%) had a scarring anus. 31/52 (60%) cases were evaluated by a previous pelvic MRI. 7/52 (13%) patients had a stoma when the stem cell therapy was performed.

Regarding the 42 perianal fistula cases, 26 cases were transsphincteric (Parks type 2) and 16 were suprasphincteric (Parks type 3) [5]; 18 of them were Crohn's-associated perianal fistulae and 24 of them presented a complex non-Crohn's fistulae (cryptoglandular fistula). Based on the information obtained by the surgeon in the operating room and the pelvic MRI when available, 37/42 (88%) cases had 1 main fistulous tract, 3/42 (7%) had 2 main tracts, and 2/42 (5%) had 4 tracts. Regarding the 7 cases of rectovaginal fistula, 3 were Crohn's related and 4 were not. Perianal and rectovaginal fistula had all multiple previous surgeries that are described in Table 1.

Stromal vascular fraction (SVF) was used in 31/52 (60%) cases, autologous expanded adipose-derived stem cells (Au-eASC) were employed in 9/52 (17%), and allogenic expanded adipose-derived stem cells (Allo-eASC) were employed in 12/52 (23%). Doses of ASCs implanted were heterogenous, being the mean 48 million, ranging from 1 to 210 million.

Both eASC (autologous and allogenic expanded adipose-derived stem cells) and SVF protocols were authorized by our hospital (University Hospital Fundacion Jimenez Diaz) and by the Spanish Medical Agency, according to European Medicine Agency (EMA) guidelines. All patients signed a detailed consent form prior to any intervention, which included permission for data publication. All ethical standards were in accordance with those of the Helsinki Declaration (1975).

### 2.1. Cell Management

#### 2.1.1. SVF from Lipoaspirate

The liposuction was performed by a plastic surgeon and obtained 80-100 ml of fat. Phosphate buffered saline (PBS; Gibco of Thermo Fisher Scientific, Waltham, MA, United States) was used to wash the raw lipoaspirate and remove local anesthetics and cells. To extract the cellular fraction, we used in 16 patients Cellution system (Cytori Therapeutics, San Diego, CA, US), in 13 patients GID system (Karam Medical, Barcelona, Spain), and in 2 patients ADSC system (Lyposmol Biotech, Zaragoza, Spain) according to manufacture instructions in all system.

Before injection, in any case in less than 2 hours, cells were suspended in sterile ringer-lactate solution (Grifols S.A., Barcelona, Spain). Phenotypic analyses and count of cells obtained were performed previously to send cell to surgery room. The cell viability was always >95% as determined by trypan blue (Sigma-Aldrich, St Louis, MO, United States).

#### 2.1.2. Autologous Expanded Adipose-Derived Stem Cells

Raw lipoaspirate product was sent in sterile bags in container at 4°C as follows: in 2 cases to the manufacturing facility of Hospital Gregorio Marañón (Madrid, Spain; manufacturer authorization no: AEMPS-20090211-TA); in 1 case to the manufacturing facility of Hospital Niño Jesús (Madrid, Spain; manufacturer authorization no. AEMPS-ES/048I/18); and in 6 cases to the manufacturing facility of Clínica Universitaria de Navarra (Pamplona, Spain; manufacturer authorization no: AEMPS-ES/127/17); in all cases according to Spanish and European legislation.

Using different authorized procedures, cell cultivation and expansion continued until the required number of cells for implantation (dose) was obtained in each manufacturing facility. For administration, the cells were suspended in a sterile-balanced saline solution with 1% human albumin, of different laboratories in each manufacture facility, at 2 × 106 cells/2 ml in all cases. To examine cell viability, DNA stability, and pathogen controls (analysis performed by the producer), samples were taken before release.

#### 2.1.3. Allogenic Adipose-Derived Stem Cells

In 6 treatments, cells were manufactured from healthy donors by the manufacturing facility of Clínica Universitaria de Navarra (Pamplona, Spain) and in 6 treatments from healthy donors by the manufacturing facility of Hospital Gregorio Marañón (Madrid, Spain). In all cases, cell viability, DNA stability, and pathogen controls were taken before packing the cells for their application. Cells were sent with temperature control at 2 × 10^6^ cells/2 ml of sterile-balanced saline solution with 1% human albumin in all cases.

All expanded ASCs were injected in less than 24 hours from delivery.

### 2.2. Treatment Procedure and Evaluation of Healing

All surgical procedures were performed at the University Hospital Fundacion Jimenez Diaz (Madrid), by the same team of surgeons, belonging to the Colorectal Surgery Unit.

Surgical technique performed was curettage, closure of the internal opening (IO), and ASC injection in 42/52 (81%) cases; curettage, endoanal flap, and ASC injection in 6/52 (11.5%) cases; and curettage, vaginal or Martius [23] flap, and ASC injection in 4/52 (7.5%) cases. Fibrin glue (Baxter Inc., Deerfield, IL, United States) enriched with ASCs was employed in surgery in 32/52 (61.5%) cases. ASC suspension was injected around the internal opening and into the tract walls through a long fine needle. The injections were superficial, not deeper than 2 mm. Surgical technique was recently described in detail and published [24].

### 2.3. Patients' Follow-Up and Outcome Evaluation

The first evaluation was performed around the 4th week in all the patients available and every 2 months until at least the 6th postoperative month.

Initial response was recorded in weeks, and the healing or not was determined at the 6th postoperative month. Patients were followed at the office every 3 months thereafter, with a mean time follow-up of 20 months (range: 6-48 months). Improvement or partial response was defined as closure of 50% of external orifices or 50% decrease of suppuration from the external orifices. Healing was defined as no suppuration from the external orifices after at least 6 months of follow-up. Relapse or reopening of the fistula was evaluated in all patients followed at least for 12 months (38/52 patients, 73%), at one year of follow-up visit. These intervals of time for follow-up were selected according to the knowledge obtained from the different stem cell CTs developed so far, and it has been modified along time [20, 21, 25, 26].

## 3. Results

Considering all the cases treated, 49/52 (94.2%) showed healing or improvement/partial response, starting in a mean time of 6.5 weeks (range: 2-36 weeks). Healing was achieved in 24/52 (46.2%) patients. Most of them were cured in a mean time of 5.5 months (range: 0.5-24 months). Healing in these 52 patients related to the type of cell used was evaluated. SVF had a curation rate of 14/31 (45.1%) cases, Au-eASC 3/9 (33.3%) cases, and Allo-eASC 7/12 (58.3%) (*p* = 0.5). Administered cell dose was also analyzed, having a mean of 41.1 million (range: 3-210 million) in cured cases and 54.1 million (range: 1-200 million) in the cases that failed to cure.

When the 42 perianal fistula cases were selected, 40/42 (95.2%) showed healing or improvement/partial response, starting in a mean time of 6.6 weeks (range: 2-36 weeks). Healing was found in 22/42 (52.4%) cases. Most of them were cured in a mean time of 5.8 months (range: 0.5-24 months). Healing in these 42 patients related to the type of cell used was evaluated. SVF had a curation rate of 13/23 (56.5%) cases, Au-eASC 3/9 (33.3%) cases, and Allo-eASC 6/10 (60%) (*p* = 0.42). Administered cell dose was also analyzed in perianal fistulas having a mean of 43.9 million (range: 3-210 million) in cured cases and 71.4 million (range: 1-200 million) in the cases that failed to cure.

If we consider only perianal Crohn's-related fistula [18], 18/18 (100%) showed healing or improvement/partial response, starting in a mean time of 5.3 weeks (range: 2-12 weeks). Healing was found in 10/18 (55.5%) cases. Most of them were cured in a meantime of 6.5 months (range: 0.5-24 months).

In the cases of perianal cryptoglandular fistula [23], 18 had curettage, closure of the internal opening, and ASC injection and presented a healing of 9/18 (50%) and 6 undergone a curettage, endoanal advancement flap, and ASC injection and turned out in fistula closure of 3/6 (50%) (*p* = 1). In all the perianal Crohn's-related fistula cases [18], the surgical technique performed was curettage, closure of the internal opening, and cell injection.

In Table 2, improvement and healing results are summarized, considering all cases treated, and different types of perianal fistula (Crohn's related or not) and its relationship to the implanted ASC type.

Regarding the rectovaginal fistula cases [7], 6/7 (85.7%) showed improvement, but none of the cases was cured after 6 months of follow-up. Most of the cases [6] received SVF and 1 case Allo-eASC.

In 3 cases of perianal fistula and 2 of rectovaginal fistula, a second dose of ASCs was administered, and healing was achieved in 3/3 (100%) cases of perianal but none of the rectovaginal fistula (Table 3).

Fibrin glue use was not related to any significant improvement in healing rates when analyzed either in the entire group or in subgroups.

In those patients followed for at least 12 months, 1 year (38/52 patients, 73%) nonrelapse of the fistulas was recorded (0%).

Although 13/52 (25%) of the cases presented fecal incontinence at the moment of enrollment, none of them had worsening throughout the follow-up period.

No adverse reactions or complications related to stem cell therapy were reported during the study period.

## 4. Discussion

An ASC compassionate use program is justified in several situations: patients who do not meet criteria of CTs in development, those who are not allowed to be enrolled for the same reason as remoteness (for example foreign patients), patients previously included in a CT control arm, or those cases included in treatment arms CT which failed to heal. As an international referral center, we developed a compassionate use program in order to expand the stem cell therapy to all the patients who can benefit from it. On the other hand, being a compassionate use program does not exempt our team from meeting all legal and ethical requirements; therefore, for each patient, an individual request to the Spanish Agency for Medicines and Health Products (AEMPS) was performed and approved (Royal Spanish Decree 1015/2009, 19^th^ of July), and consent forms were signed by both the attending surgeon and the patient.

Although the present study had the limitation of including a heterogenous population which is treated without a standard surgical technique, we still can get important findings.

We treated 52 patients with chronic nonhealing diseases, achieving an improvement in more than 90% of the cases and a mean complete healing rate of 46% ranging from 44 to 69% (according to different bundling of cases) at 6-month follow-up. Perianal fistula, cryptoglandular fistula, and Crohn's-related fistula showed a healing rate of 52.4%, 50%, and 55.5%, respectively. These results are consistent with previously published CTs [20, 21] considering most of these patients received only one dose of ASCs.

Regarding perianal Crohn's-related fistula, we treated 13 patients; they received a first dose of ASC and 5 of them were healed. Three patients received a second dose and healed all of them. Later, 2/13 patients received a second dose to treat another fistula, different to the one already cured, and they both healed. Therefore, 53% of the patients were cured after a first injection of ASC, and the patients treated with a second ASC injection were cured 100%. As we did not treat all patients with a second dose, we cannot assert but we can estimate a healing increase around 30% with the second dose of ASCs, which is consistent to previously published results [19, 20]. Five of the 13 Crohn's patients were not cured; 1 is waiting for a second ASC treatment, 3 are enrolled in a new ASC CT, and 1 refused a second dose of stem cells.

Rectovaginal fistula, both Crohn's related or not, is one of the most difficult to treat and achieve healing. In this study, we treated 7 cases and none of them was cured. Six cases were treated with SVF and one with Allo-eASC. One patient was treated with a first dose of SVF and a second injection of Allo-eASC and another patient with 2 doses of SVF; the rest of the patients were treated only once using SVF. This result is not very congruent to previous data published by our group but can be explained due to the extreme complexity of these clinical cases. Although at that time, we reported 60% of closure rate; the difference could be explained by administration of up 2 doses of allogenic ASCs in all patients in that study [27]. On the other hand, for the compassionate use program, SVF was the main treatment. Therefore, the lower dose of stem cells and the autologous use could be an explanation for the results.

No statistical relationships have been established between the use of fibrin glue, surgical approach, cell lineage, or doses used, due to the small number and variability of patients, but some patterns can be observed to guide in the future the best use of ASCs to treat chronic nonhealing disease.

After one year of follow-up, there were no relapses or associated fecal incontinence cases.

ASC use in the treatment of nonhealing disease and fistula disease is a safe procedure; we did not record any adverse event, not even perianal abscesses.

Contrary to a CT, in a compassionate use program, the cell growth protocols are not unified; for example, we received expanded ASC cells cultivated in fetal bovine serum and others in lysed platelets, although both protocols are approved by regulatory health agencies. On the other hand, when using SVF, we observed healing rates similar and sometimes superior to the expanded ASC; this idea seems inconsistent to previous publications [19], where a superior anti-inflammatory process control is described for expanded ASC better than for SVF. It is possible that the transportation system and temperature control have an effect in the expanded cells that we will have to figure out, since SVF is produced at our institution, is injected in less than 2 hours, and consequently is not exposed to these factors. Therefore, several issues make our data less homogeneous than if they were obtained through a CT. On the other hand, the low number of cases precludes to stablish a relationship to growing or transporting protocols and healing rates.

We can summarize that the weakness of this study is the significant heterogeneity of the examined population, the unstandardized surgical techniques, and the heterogeneity of used stem cells including the source and dosage of cells.

In conclusion, the use of ASCs in the treatment of perianal fistula is a minimally invasive procedure and safe technique that lacks impairment of fecal continence, unlike other surgical techniques as endoanal advancement flap that could reach a fecal incontinence rate up to 30% [27, 28]. In addition, repeated doses of ASC injection improve the rate of healing, without adding adverse effects. When treating perianal Crohn's-related fistula, this issue is more relevant. We try to avoid complex fistula anal techniques (endoanal advancement flap and others) because of the increased risk of fecal incontinence; in these patients, ASC injection gains relevance and increases the healing rate around 17% to the standard surgery with curettage and seton management [21]. Finally, having a compassionate use program allows our team to offer ASC therapy to all the patients who can benefit from it.

## Figures and Tables

**Table 1:** Previous surgeries (type) in perianal and rectovaginal fistulas.

Type of fistula	I&D^∗^	I&D&S^∗∗^	Endoanal flap	Vaginal/Martius flap	I&D and ASCs	Others^∗∗∗^	Endoanal flap and others
Perianal fistula	2	31	2	0	5	1	1
Rectovaginal fistula	0	2	0	5	0	0	0

^∗^I&D: incision and drainage; ^∗∗^I&D&S: incision and drainage and multiple seton placement. ^∗∗∗^Others: FILAC, PLUG.

**Table 2:** Healing related to the type of implanted ASC.

Cases	*N*	Improvement (%)	Healing (%)	SVF healing (%)	AU-eASC healing (%)	Allo-eASC healing (%)
All cases	52	94.2	46.2	45.1	33.3	58.3
All perianal F^∗^	42	95.2	52.4	56.5	33.3	60
Perianal Crohn's F	18	100	55.5	40	66.6	55.5
Perianal cryptoglandular F	24	91.6	50	69.2	16.6	40

^∗^F: fistula; all *p* > 0.05.

**Table 3:** First and second doses in the same case.

Patient ID	Fistula type	Crohn's	Dose number	ASC type	Number of millions	Healing
1	Suprasphincteric	Yes	First	SVF	6.5	No
1	Suprasphincteric	Yes	Second	SVF	8	Yes
2	Rectovaginal	No	First	SVF	2	No
2	Rectovaginal	No	Second	Allo-eASC	42	No
3	Transsphincteric	Yes	First	SVF	13	No
3	Transsphincteric	Yes	Second	Au-eASC	40	Yes
4	Rectovaginal	Yes	First	SVF	15	No
4	Rectovaginal	Yes	Second	SVF	1	No
5	Suprasphincteric	Yes	First	SVF	6.5	No
5	Suprasphincteric	Yes	Second	Au-eASC	42	Yes

## Data Availability

All the data were gathered from our institution database and cannot be shared due to confidentiality issues.
